# Interventional Approach to Portal Vein Thrombosis and Liver Transplantation: State of the Art

**DOI:** 10.3390/life13061262

**Published:** 2023-05-26

**Authors:** Vijay Ramalingam, Lauren M. Yang, Colin J. McCarthy, Muneeb Ahmed

**Affiliations:** 1 Division of Vascular and Interventional Radiology, Beth Israel Deaconess Medical Center, Harvard Medical School, Boston, MA 02215, USA; 2 Division of Hepatology and Gastroenterology, Beth Israel Deaconess Medical Center, Harvard Medical School, Boston, MA 02215, USA

**Keywords:** portal vein thrombosis, portal vein recanalization, transjugular intrahepatic portosystemic shunt, liver transplant

## Abstract

Porto-mesenteric vein thrombosis (PVT) is a well-recognized but uncommon disease entity in patients with and without cirrhosis. Given the complexity of these patients, there are many differing treatment algorithms depending on the individual circumstances of a given patient. The focus of this review is primarily patients with cirrhosis, with an emphasis on liver transplantation considerations. The presence of cirrhosis substantially affects work-up, prognosis, and management of these patients and will substantially affect the patient treatment and have additional implications for prognosis and long-term outcomes. Here, we review the incidence of portal vein thrombosis in known cirrhotic patients, medical and interventional treatment options that are currently used, and, in particular, how to approach cirrhotic patients with PVT who are awaiting liver transplantation.

## 1. Introduction

Porto-mesenteric vein thrombosis (PVT) is a well-recognized, albeit uncommon disease entity in patients with and without cirrhosis [1,2,3,4,5]. The focus of this review will primarily discuss patients with cirrhosis, with an emphasis on liver transplantation considerations. Because the presence of cirrhosis substantially affects work-up, prognosis, and management, it is important in all patients to determine whether cirrhosis is present at the time of initial diagnosis of acute or chronic PVT. This will substantially affect the next steps in work-up and treatment and have additional implications for prognosis and long-term outcomes [1,2]. Here, we review the incidence of portal vein thrombosis in known cirrhotic patients, medical and interventional treatment options that are currently used, and in particular, how to approach cirrhotic patients with PVT who are awaiting liver transplantation.

## 2. Incidence and Etiology of PVT in Cirrhotic Patients

The risk of portal vein thrombosis in patients with cirrhosis is estimated to be approximately 10% in patients with compensated cirrhosis, with increasing prevalence in more advanced stages of cirrhosis. The rate of PVT is as high as 26% in decompensated liver transplant candidates [6,7,8,9]. The pathophysiology of developing PVT in the setting of underlying cirrhosis is related to slow portal blood flow due to increased intravascular resistance, together with stasis, and alterations in the balance of pro-coagulant and anti-coagulant clotting factors produced by the liver. Common cirrhosis-related hemostatic derangements include decreased anticoagulant factors such as protein C and increased prothombotic factors such as factor VIII and thrombin [10]. Additional risk factors that may contribute to a pro-thrombotic portomesenteric state include diabetes, obesity, and a history of prior abdominal surgery, such as splenectomy, where post-operative vascular anatomy may lead to altered mesenteric flow dynamics [11]. With more advanced cirrhosis, a combination of these factors results in higher risk of PVT. 

The presence of HCC is also associated with development of PVT, not only due to direct vascular invasion (tumor thrombus), but also related to the prothrombotic nature of cancers, including HCC, in general with patients expressing thrombophilic genetic factors at higher risk than those without [12,13]. It is important to differentiate HCC tumor thrombus from bland portal vein thrombosis due to the differing treatments between the two entities. Additionally, patients with cirrhosis who experience an episode of acute pancreatitis are at higher risk of development of PVT than patients without cirrhosis, with the presence of cirrhosis being a risk factor for higher inpatient mortality. This is likely due to complications related to portal hypertension such as gastrointestinal bleeding, rather than those related to pancreatic inflammation and systemic inflammatory response syndrome (SIRS) [14]. 

## 3. Impact of Pre-Transplant PVT on Transplant Outcomes

Liver transplantation is a mainstay treatment for patients with decompensated liver cirrhosis and other liver conditions with high mortality rates. At five years, post-transplantation survival rates can exceed 75% in well-selected patients [15]. Earlier studies that examined outcomes in transplanted patients demonstrated an increase in early (90-day) and late (one-year) graft failure and patient mortality in those patients with pre-operative PVT [16]. Additionally, one study noted that five-year graft survival varied from 88.3% in those patients without PVT to 47.7% in patients with PVT [17]. Because the graft is highly dependent on portal flow for perfusion and oxygenation, any degree of PVT was historically considered a contraindication to liver transplantation in many centers. Complex chronic portal vascular changes still remain a barrier to transplantation at many centers [18,19]. Additionally, there are data suggesting that the mere presence of complete portal vein thrombosis increases the risk of death 30 days and 1 year post-transplantation [20,21,22]. 

**Figure 1:**
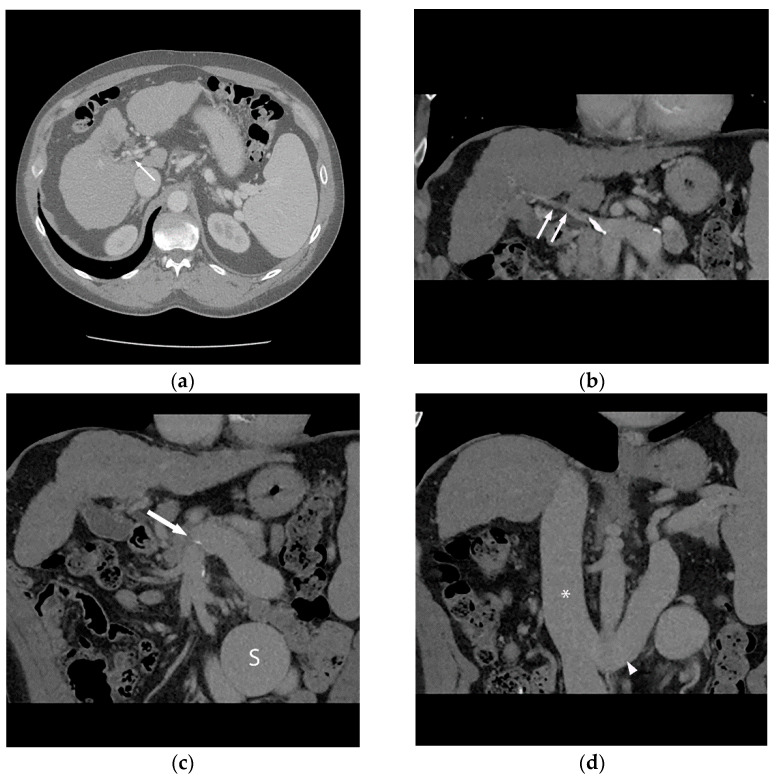
56-year-old male with history of alcohol-related cirrhosis and portopulmonary syndrome. His baseline values included MELD-Na 19 (Tbili 4.0, INR 2.0, Cr 1.0, Na 137). He had imaging evidence of non-occlusive main portal vein thrombosis. Axial (**a**) and coronal (**b**) contrast-enhanced CT shows a diminutive portal vein (arrow). Coronal contrast-enhanced CT (**c**,**d**) demonstrates this extended to the portal venous confluence (arrow), with partially visualized component of a large inferior mesenteric vein to gonadal vein portosystemic shunt (S), which drained via the renal vein (arrowhead) to the inferior vena cava (*).

Portal vein thrombosis impacts liver transplant outcomes in several ways. First, the presence of acute or chronic portal vein thrombosis (Figure 1) reduces the likelihood of achieving a physiologic portal-to-portal anastomosis of the donor and recipient portal vein, which leads to worse outcomes and increased mortality amongst liver transplant recipients [17,19,20]. This has downstream impacts of requiring the creation of alternative surgical venous anastomoses, which have generally limited retrospective reports of success and long-term outcomes, and in some instances, altered portal perfusion and/or reduced contributions from the mesenteric system reduce overall liver function, graft survival, etc. Second, intraoperative strategies to address PVT increase overall operative times. These techniques include portal vein surgical thrombectomy or the creation of alternative shunts, which can affect graft viability through prolonged cold and warm ischemia times (both themselves predictors of graft viability) [16]. Adequate graft perfusion in the immediate peri-operative period is critical to successful outcomes after liver transplantation [2,21]. Third, the presence of advanced PVT at the time of transplant is associated with a higher incidence of recurrent post-transplant PVT, itself a predictor of greater post-transplant morbidity, longer ICU times and hospital stays, and higher rates of graft loss [22]. Finally, there have been some recent data suggesting a higher incidence of biliary leaks and strictures in the post-operative state in patients with pre-operative PVT during liver transplantation [17]. 

The impact of portal vein thrombosis on liver transplantation has not been fully appreciated, given that until recently PVT was a contraindication or relative contraindication to liver transplantation, and data on non-listed patients were incomplete. Determining the impact of portal vein thrombosis in liver transplantation is further complicated by the fact that until recently, pre-operative incidence of PVT in patients ultimately not listed for transplant has not been well-documented. In fact, one review estimated that the true incidence of portal vein thrombosis amongst liver transplant candidates may be as high as 44% [21]. Furthermore, the influence of PVT on liver transplant listing decisions in patients has been poorly documented [7]. Thus, the additional impact of both underestimated PVT incidence in this population and the downstream increased mortality associated with the decision to not offer a liver transplant is likely also underrepresented in studies evaluating the impact of portal vein thrombosis in liver transplant candidates [8,21]. While there are many unanswered questions, it is clear that portal vein thrombosis increases the surgical complexity of liver transplantation and likely affects the immediate and long-term survival of patients diagnosed with PVT in the pre-operative setting [20,22]. 

## 4. Efficacy of Medical Management in Portal Vein Thrombosis

Anticoagulation remains a mainstay of PVT treatment, with studies suggesting that anticoagulation treatment prevents thrombus progression, can result in thrombus improvement, decreases variceal bleeding, and improves ascites [2]. Current guidelines from the American College of Gastroenterology and American Association for the Study of Liver Disease both endorse anticoagulation treatment as a mainstay of therapy in patients with PVT being considered for the possibility of liver transplantation due to the deleterious effects progressive thrombus burden [23,24,25]. One proposed definition for ‘potential candidate for liver transplantation’ is all patients with cirrhosis without a definitive contraindication to liver transplantation [26]. There is also consensus that patients initiated on anticoagulation within the first six months of diagnosis and with partial PVT are more likely to benefit from the use of anticoagulation [27]. Currently, there is a significant increase in portal vein recanalization rates with the addition of anticoagulation rather than observation, but only 42% of patients demonstrate complete recanalization of the portal vein [24]. Complicating this, spontaneous resolution of PVT can be seen in up to 40% of patients with partial PVT [28]. Finally, regardless of the strategy used, up to 40% of patients will re-thrombose their portal vein after initial resolution of PVT within two to five months following completion of systemic anticoagulation [2,21,29]. Regardless of anticoagulation strategy, it is clear that there are a substantial number of patients who will not benefit from anticoagulation therapy at all or will re-thrombose shortly after completion of anticoagulation therapy. Additionally, there are a number of patients who cannot tolerate anticoagulation for a variety of cirrhosis-related reasons. Furthermore, there still remain multiple questions (and opinions) on which patients are most suited to initiation of anticoagulation as first-line therapy, the duration of therapy before treatment non-response or partial response is determined, and the definition of response to treatment (partial vs. complete response)—with most consensus statements ultimately deferring to center expertise. 

## 5. Surgical Options and Intraoperative Management of PVT during Liver Transplantation

Achieving a physiologic portal vein-to-portal vein anastomosis is the optimal surgical outcome during liver transplant, which results in similar overall outcomes between patients with PVT and those without [7]. The key underpinning principle is that sufficient mesenteric venous blood is returned to the liver, which is imperative for transplant graft function and also should support sufficient venous flow so as to minimize the risk of post-operative portal vein thrombosis. 

To that end, there has been increasing focus on alternative surgical management strategies that focus on the restoration of physiologic portal flow even in the absence of a portal end–end anastomosis defined as restoring splanchnic blood flow to the liver graft in order to relieve the effects of portal hypertension [30]. Currently, the ideal surgical strategy in patients with PVT is either to perform a physiologic end–end anastomosis or, if there is too much thrombosis present, perform a venous eversion with sharp dissection of the thrombus until there is sufficient vessel lumen to perform the anatomic anastomosis [21]. In more complicated PVT patients where that strategy is not able to be performed, several additional strategies have been performed and reported [21,30]. These include donor portal vein to recipient super mesenteric venous anastomosis or, in the presence of thrombus at or near the superior mesenteric vein, interposition or jump grafts maybe used to complete the anastomosis [21]. Additional surgical options to restore physiologic inflow include the use of a coronary vein or large collateral vein as the anastomotic site or the use of a large spleno-renal shunt to create a reno-portal anastomosis [21,30]. Other options that have been described for surgical management of portal vein thrombosis include porto-caval hemitransposition as well as portal vein arterialization; however, both of these strategies have been considered suboptimal due to the non-physiologic nature of the anastomosis and remaining portal hypertension [30,31,32]. Regardless of the solution, ultimately surgical options for management of portal vein thrombosis intraoperatively are still limited by a lack of data and predominantly described in case-series or case reports. As such, these techniques are often based on center-specific expertise and have not received widespread adoption, which continues to influence whether patients with underlying known PVT are even considered for liver transplant.

## 6. Classification Scheme and Impact on Transplantation

There have been multiple classification schemes (Table 1) devised to categorize portal vein thrombosis [2]. In fact, to date there have been nine proposed schemes to categorize PVT [30,31,32,33,34,35,36,37]. While all of the classification systems except one utilize four grades of thrombosis, there is otherwise no relation between the different systems. Of the proposed systems to grade and categorize portal vein thrombosis, only the Yerdel, Charco, and Jamieson system have relevancy when it comes to transplantation and surgical management due to the fact that these grading systems take into account the extent of thrombosis in a manner that will dictate surgical decision making [30,35,36,37]. The Yerdel system, which has now become the most widely used system, defines only the extent of the thrombosis and does not account for large collaterals that may be used in transplantation [30,35]. The Jamieson and Charco system similarly accounts for the degree and extent of thrombosis in order to aide in pre-operative surgical decision making to both achieve an end–end portal–portal anastomosis (or in a manner that will restore physiologic inflow), and also take into account the presence or absence of large collateral vessels [30,36,37]. The Bhangui system incorporates the extent and degree of thrombosis as well as the presence of collaterals, but goes one step further by including thrombus complexity to help determine whether physiologic or non-physiologic portal flow can be restored during transplantation [30]. To that end, they categorize Yerdel I–III as non-complex due to the fact that end-to-end anastomosis or thrombectomy with jump-graft can be performed at surgery [30]. By contrast, Yerdel grade 4, and advanced grades of the Jamieson and Charco schema (grades 3 and 4), would be considered complex PVT due to the fact that the surgical strategy would have to change in order to facilitate liver transplantation [30]. Overall, these differences underscore the lack of consensus and evolving understanding of the factors that affect the potential to achieve a physiologic portal inflow and their impact on long-term outcomes after transplant. Furthermore, none of these systems incorporate the availability of newer interventional techniques described below, which have a different set of criteria for patient selection and predicting technical success. Additionally, newer systems will need to take into account that newer interventional techniques may offer solutions for patients with recent and chronic thrombosis as well as thrombosis involving multiple segments. Ultimately, the optimal classification scheme will need to incorporate all of these elements, and then be validated in prospective studies across multiple centers. 

## 7. Role of Endovascular Recanalization of Portal Vein

In light of the suboptimal results of anticoagulation alone as well as the concerns for poor outcomes in transplantation patients, there has been increased interest in endovascular options for portal vein recanalization [38]. While endovascular options including transhepatic thrombolysis as well as portal vein stenting have previously been evaluated, in the setting of pre-transplantation patients, transjugular intrahepatic portosystemic shunt (TIPS) creation is the most widely utilized intervention given the ability to restore portal vein patency with the goal of providing the surgeon an adequate native vein to create an end-to-end anastomosis. Endovascular portal vein recanalization (PVR) for PVT patients utilizing TIPS has been reported for over decades, when it was first reported in the management of variceal bleeding [39]. It has since been reported on and studied for its potential use for portal vein thrombosis in cirrhotic and non-cirrhotic patients with or without the addition of anticoagulation [38,40,41]. More recently, the use of PVR-TIPS has been reported specifically in pre-transplantation patients given the ability to restore portal vein patency to allow a traditional transplant surgery without the need for increasing the complexity of transplant utilizing non-traditional anastomotic techniques such as renoportal anastomosis or portal vein arterialization [42,43,44]. While there are varying reports of success with heterogeneous study populations, the overall success rate of portal vein recanalization with TIPS creation is reported to be 95–98%, with most studies reporting an acceptable complication rate consistent with previously reported TIPS literature [42,45]. 

## 8. Performing PVR-TIPS Prior to Liver Transplant

There are several described techniques for portal vein recanalization in patients with portal vein thrombosis. For purposes of this review, those interventions specifically relevant to pre-transplantation patients will be described. 

Pre-procedure assessment and planning. Prior to any procedure, all patients should have an appropriate evaluation by an interventional radiologist, transplant surgeon, and a hepatologist to ensure both adequate transplant candidacy and optimization of liver function and medical therapy prior to proceeding with PVR-TIPS. A decision to proceed should take into account current liver transplant eligibility and listing status (including estimated waiting time in the region), and whether the patient has additional symptoms (e.g., high-risk varices or refractory ascites) that might warrant earlier intervention. All patients should have updated clinical testing, including liver function tests, coagulation assessment, a MELD-Na score, and Child–Pugh scores. In particular, in patients who have more advanced liver dysfunction, including MELD-Na scores > 18–21 or advanced Child–Pugh scores (B or C groups), ensuring that patients are eligible for and/or are actively listed for a liver transplant is important in the event of hepatic decompensation following a procedure. Recent cross-sectional imaging should also be obtained prior to evaluation, with either a contrast-enhanced multiphasic CT or an MRI, to delineate the extent of the thrombosis. Finally, patients receive an echocardiogram to assess cardiac function prior to intervention. At our center, procedures are all performed with general anesthesia and followed by inpatient admission for close post-procedure monitoring. 

**Figure 2:**
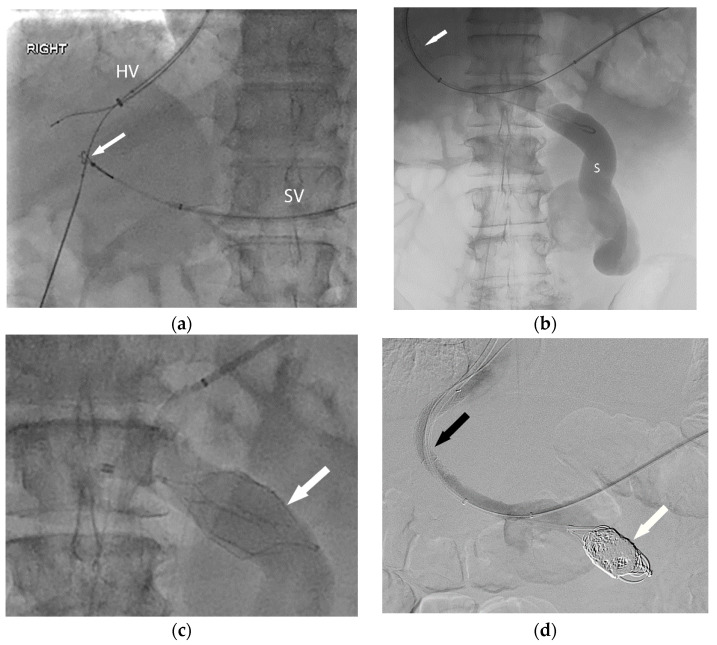
In order to facilitate listing for liver transplantation, the patient (Figure 1) was referred for portal vein recanalization and transjugular intrahepatic portosystemic shunt (TIPS) placement. (**a**) This required both hepatic vein (HV) and splenic vein (SV) access, and a “gun-sight” technique (arrow) was used to reconstruct the portal vein. (**b**) With the TIPS deployed (arrow), the large shunt (S) was next addressed. (**c**) An inferior vena cava filter (arrow) was used in an off-label manner to form a scaffold for coil embolization of the large portosystemic shunt. Final angiography (**d**) demonstrates a patent TIPS (black arrow) and occluded portosystemic shunt (white arrow).

PVR-TIPS techniques: The ability to successfully perform a PVR-TIPS requires mastery of several core building-block techniques that are generally employed in step-wise fashion (Figure 2). First, the steps of a standard TIPS placement procedure should be performed, including obtaining right internal jugular vein access, placement of a 10 Fr sheath, obtaining a baseline right atrial pressure measurement, and catheterization of the right hepatic vein [42,43,44]. Once in position, the cannula device from a TIPS kit is then placed within the sheath, and in cases of partially or minimally occlusive thrombosis, access into the portal vein is obtained utilizing standard techniques. In complete or chronic occlusion (with or without cavernous transformation), transhepatic or transplenic techniques are utilized to obtain access into the thrombosed portal system.

**Figure 3:**
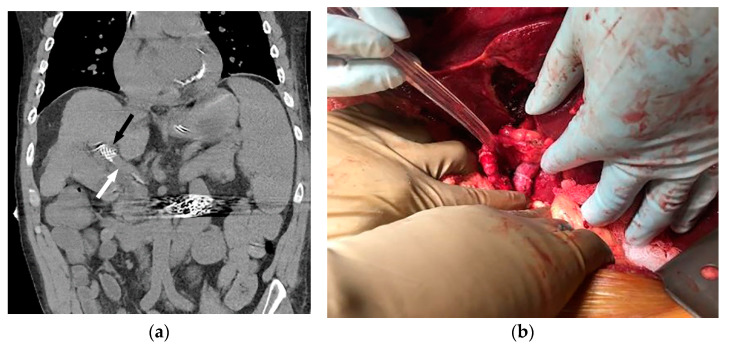
Coronal non-contrast CT (**a**) performed two days later (same patient as Figure 1 and Figure 2) demonstrates a larger-caliber portal vein (white arrow). The caudal aspect of the TIPS is noted (black arrow). The patient underwent successful orthotopic liver transplant approximately 1 year later (**b**), and remains well after 53 months of follow-up.

Initial series described transhepatic access to gain access into the thrombosed portal system; however, more recent series described a higher technical success rate for the procedure with the use of transplenic access into the portal system [43], which has been the experience at our center. In cases where transplenic access is unfavorable or unable to be employed, transmesenteric access has also been utilized as a possible method for portal vein recanalization [46]. In the case of transplenic access, a 21 G needle is advanced under ultrasound guidance into an intraparenchymal splenic vein branch and a 0.018 inch nitinol wire is advanced through the needle into the splenic vein. At this point, an Accustick sheath (Boston Scientific Corporation, Marlborough, MA, USA) is placed into the splenic vein and a long digital subtraction image of the portal venous system is obtained and portal pressures are obtained. Oftentimes, a faint diminutive caliber thrombosed portal vein can be identified coursing superiorly towards the liver hilum, and at this point utilizing an angled catheter and glide wire the portal vein is recanalized and a small amount of contrast is injected to confirm intrahepatic portal vasculature. Even in cases with extensive chronic thrombosis with cavernous transformation, a long DSA run will often opacify the obliterated portal vein or identify the coronary vein, which can be used as a landmark to identify the chronically thrombosed segment. Once access is confirmed, the main portal vein is dilated with an 8 mm high-pressure balloon to restore patency and facilitate TIPS access and placement. After balloon dilation, TIPS is performed via the standard technique via the right internal jugular access, and in the event of difficult access into the portal system, a snare is placed via the transplenic access in order to help target the access site and obtain through and through access. After obtaining portal access and measuring the tract length, stent deployment is carried out in a fashion so as to maximize the amount of unstented portal vein in order to facilitate end-to-end anastomosis during surgery (Figure 3). Additional considerations during stent placement include ensuring that the superior portion of the stent is not extended into the inferior vena cava or right atrium or that the inferior portion of the stent does not extend into the retropancreatic region. Additionally, it is worth noting that stent placement is highly center- and surgeon-dependent so these discussions should take place prior to the procedure. After stent deployment, the entirety of the stent and parenchymal tract is dilated with a 10 mm high pressure balloon and in the event of recalcitrant stenosis or thrombosis, a 12 mm high pressure balloon. Post-TIPS DSA images are obtained and in the event of large collateral vessels influencing portal vein and TIPS flow, these are embolized at the time of the procedure to maximize portal vein flow. At the conclusion of the procedure, post-TIPS creation main portal and right atrial pressures are obtained. The splenic access site may be closed utilizing gelfoam pledgets, although prior operators have reported the use of coils with high technical success [44]. 

## 9. Early Clinical Outcomes

Early clinical outcomes of portal vein recanalization for pre-transplantation purposes demonstrate that the procedure is technically safe with the most common complications being TIPS stenosis or medically treatable hepatic encephalopathy [43]. More serious complications such as bleeding or right heart failure have been reported but are rare in appropriately selected patients [43]. The initial large series reported by Salem et al. in 2015 described this pre-transplant portal vein recanalization procedure being technically successful in 60/61 patients with preserved portal vein patency at 16.7 months of follow-up in 55/60 patients [42]. At the time of publication, 23/60 patients went on to successful transplantation, of which 22/23 were able to achieve a standard end–end portal vein anastomosis [42]. Of note, the one patient who did not receive a standard end–end anastomosis went on to receive an interposition graft and thus was able to obtain physiologic inflow. At the time of their initial report, there were no reported cases of post-transplant portal vein thrombosis in the 23 patients who were transplanted. Further outcomes on this cohort were published approximately two years later, where 55/60 patients who were successfully recanalized and maintained portal vein patency throughout their follow-up or until transplantation for a mean follow-up of 19.2 months [43]. Of note, of the patients who did not go to transplantation and were only followed by imaging, they maintained patency for a mean follow-up time of 25.2 months [43]. The final analysis showed that 24/60 patients went on to successful transplantation, of which 23/24 received a standard end–end anastomosis. There was no evidence of post-transplantation PVT in 24/24 patients at a medial follow-up of 32.5 months [43]. 

## 10. Developing Center Expertise—Role for Formal Multidisciplinary Care

Given the complexities of management of this patient population and the need for input from multiple specialties, our center has instituted a formal multidisciplinary splanchnic venous thrombosis program. Prior studies have validated the need for expertise in the interventional management of portal hypertension [47]. The multidisciplinary care approach has previously been studied as a method to improve survival and outcomes in other complex patient populations requiring multispecialty input such as those patients with malignancies. Additionally, it has been demonstrated that approaching patients with portal vein thrombosis with an individualized approach to treatment improves the response to therapy [38]. Formalizing a structured weekly clinic where hepatology, transplant surgery, interventional radiology, and hematology teams can discuss and evaluate every patient has ensured that each patient receives a tailored treatment algorithm that is agreed upon by all managing teams. 

## 11. Future Studies—More Outcomes Data, Better Classification, Standardized Techniques, Timing

Now that recent studies have demonstrated the safety and efficacy of portal vein recanalization techniques in addressing pre-operative acute and chronic portal vein thrombosis, additional questions remain to be answered—specifically, multi-center studies validating early studies are required, standardization of terminology and lexicon, and prospective studies with clear patient selection criteria matched to long-term peri-transplant and post-transplant outcomes. Standardization of techniques (both with regard to IR technique, but also with consensus from the transplant surgery community with regard to acceptable anatomic outcomes to guide refined IR decisions making and technique) and determination of timing of when to intervene as it relates to this patient cohort are required. Furthermore, understanding how to interleave the use of PVR-TIPS in the broader population of cirrhotic patients with portal vein thrombosis regardless of immediate transplant eligibility will be important given the evolving transplant criteria, the movement of patients across different transplant centers, and the potential for patients with portal vein thrombosis to become transplant-eligible over time. Finally, given the complexities of decision making in this population, formalizing a multidisciplinary approach to care and decision making is essential in the care of these complex patients. 

## Figures and Tables

**Table 1:** Classifications/grading of non-tumoral portal vein thrombosis (PVT) in cirrhotic patients.

Study (Reference)	Year	Study Type, Population	Grade 1	Grade 2	Grade 3	Grade 4	Important Findings
Yerdel et al. [35]	2000	Prospective, intraoperatively-confirmed PVT during OLT. 779 patients. 8.1% (*n* = 63)—operatively confirmed.	<50% of the vessel lumen with or without minimal extension into the SMV.	50–100% occlusion of the PV, with or without minimal extension into the SMV.	Complete thrombosis of both PV and proximal SMV. Distal SMV open.	Complete thrombosis of the PV, proximal and distal SMV.	Incidence of PVT was 8.1%. Grade 1 PVT patients did as well as controls. Grade 2–4 PVT associated with higher complication rate, higher in-hospital mortality, and lower long-term survival.
Jamieson et al. [36]	2000	Commentary	Thrombosis confined to the PV trunk, beyond the splenomesenteric confluence. Partial or complete.	Thrombus extending to proximal SMV.	Diffuse thrombosis of the splanchnic venous system, but with large accessible collaterals.	Extensive thrombosis of the splanchnic venous system but with only fine collaterals.	Stresses that thrombosis confined to the PV proper is comparable to that of patients without PVT. Discusses innovative surgical techniques (e.g., jump grafts) where necessary.
Charco et al. [37]	2005	Commentary	Partial or complete thrombosis, limited to PV.	Thrombosis extending to proximal SMV.	Diffuse thrombosis of the splanchnic system, with dilated collateral veins.	Diffuse thrombosis with presence of fine collateral veins.	Notes that most agree that diffuse PV thrombosis at the mesenteric confluence, without dilated veins and with enough flow for a by-pass, should not undergo transplantation.
Bhangui et al. [30]	2019	Scoping review of 9 existing classification systems.					Notes that 8 of 9 existing systems include 4 grades. No one grade common to all classification systems. Proposes classification to include Non-complex (Yerdel 1–3) and Complex (Yerdel 4, Jamieson 3–4 or Charco 3–4).

Abbreviations: SMV—superior mesenteric vein; PV—portal vein; OLT—orthotopic liver transplant; PVT—portal vein thrombosis.

## Data Availability

Not applicable.
